# A Systematic Review of the Research Development on the Application of Machine Learning for Concrete

**DOI:** 10.3390/ma15134512

**Published:** 2022-06-27

**Authors:** Kaffayatullah Khan, Waqas Ahmad, Muhammad Nasir Amin, Ayaz Ahmad

**Affiliations:** 1 Department of Civil and Environmental Engineering, College of Engineering, King Faisal University, Al-Ahsa 31982, Saudi Arabia; mgadir@kfu.edu.sa; 2 Department of Civil Engineering, COMSATS University Islamabad, Abbottabad 22060, Pakistan; waqasahmad@cuiatd.edu.pk; 3 MaREI Centre, Ryan Institute and School of Engineering, College of Science and Engineering, National University of Ireland Galway, H91 HX31 Galway, Ireland; a.ahmad8@nuigalway.ie

**Keywords:** machine learning, concrete, prediction, modeling, bibliographic data, scientometric analysis

## Abstract

Research on the applications of new techniques such as machine learning is advancing rapidly. Machine learning methods are being employed to predict the characteristics of various kinds of concrete such as conventional concrete, recycled aggregate concrete, geopolymer concrete, fiber-reinforced concrete, etc. In this study, a scientometric-based review on machine learning applications for concrete was performed in order to evaluate the crucial characteristics of the literature. Typical review studies are limited in their capacity to link divergent portions of the literature systematically and precisely. Knowledge mapping, co-citation, and co-occurrence are among the most challenging aspects of innovative studies. The Scopus database was chosen for searching for and retrieving the data required to achieve the study’s aims. During the data analysis, the relevant sources of publications, relevant keywords, productive writers based on publications and citations, top articles based on citations received, and regions actively engaged in research into machine learning applications for concrete were identified. The citation, bibliographic, abstract, keyword, funding, and other data from 1367 relevant documents were retrieved and analyzed using the VOSviewer software tool. The application of machine learning in the construction sector will be advantageous in terms of economy, time-saving, and reduced requirement for effort. This study can aid researchers in building joint endeavors and exchanging innovative ideas and methods, due to the statistical and graphical portrayal of participating authors and countries.

## 1. Introduction

### 1.1. Background

The fast global growth of data estimation techniques and analytical methods currently plays a key role in nearly every field of study [1,2]. These models and methods are enhanced using data science principles, because data science facilitates intelligent and intellectual work in several areas such as sensor-based smart farming, learning associations, prediction, weather forecasting, healthcare, etc. [3]. In addition, data sciences have permeated every aspect of tool development and have become a pillar of engineering and scientific disciplines. Furthermore, data science has spawned other subdisciplines, including artificial intelligence (AI), machine learning (ML), deep learning, etc. [4,5,6,7,8,9,10]. These subdisciplines provide a deeper grasp of learning and fundamental relationships, and allow for dealing with valuable datasets, diverse data sources, computer systems for data-concentrated functions, data privacy, and other related topics [11,12,13,14,15]. Nevertheless, ML is one of the most powerful and in-demand technologies globally [16,17,18,19,20]. Furthermore, it is a well-established tool of AI.

### 1.2. Literature Review

Generally, ML focuses on developing a computer’s or a model’s learning abilities through interpretations, prior practices, and training data samples [21,22,23]. It provides many programs and techniques for designing a learning model and for self-improvement when subjected to new datasets. ML has a vast application scope that includes data analytics, regression, prediction, classification, learning association, clustering, extraction, audio and picture recognition, etc. [24,25]. Forecasting and analysis are the best-suited application fields among these applications, where previous data are used to predict future probabilities and to provide a more precise evaluation of future probabilities [26,27]. In addition, ML is becoming an integral aspect of every subject, and researchers worldwide have focused increasingly on ML functions in each area. Recently, the infrastructure and building sector has been rapidly expanding on a global scale. ML technologies aid concrete specialists, engineers, and scientists in developing the dissemination of knowledge about their material [28]. Concrete consists of four main elements: water, fine and coarse aggregates, and cement as a binder [29,30,31]. Some supplementary elements such as fly ash, silica fume, or chemical combinations have been utilized to enhance the performance and strength of concrete [32,33,34,35,36]. The literature indicates that researchers are concentrating increasingly on these supplementary materials, since they are often waste materials created as a result of industrial, agricultural, and municipal processes [37,38,39,40,41]. The recycling and the widespread stockpiling of these leftovers for bulk use raise serious social and environmental problems on a global scale [42]. As an example, fly ash is a waste that is detrimental to the environment since it results in groundwater contamination, air pollution, and ailments in the human body [43]. Concrete strength tests are typically conducted between 3 and 28 days on samples of concrete [44,45,46,47]. The 28-day timeframe creates construction delays, although the consequences of ignoring the test would be minimal. For quality management and pre-designing in the construction industry, it is essential to create systems for the quick and accurate prediction of material strength properties. ML enables improved prediction models and methods, including decision trees, support vector machines, linear regression, random forest, regression trees, neural networks, water cycle algorithms, etc. [48,49,50,51,52,53,54,55,56].

### 1.3. Categories of Machine Learning

**Figure 1:**
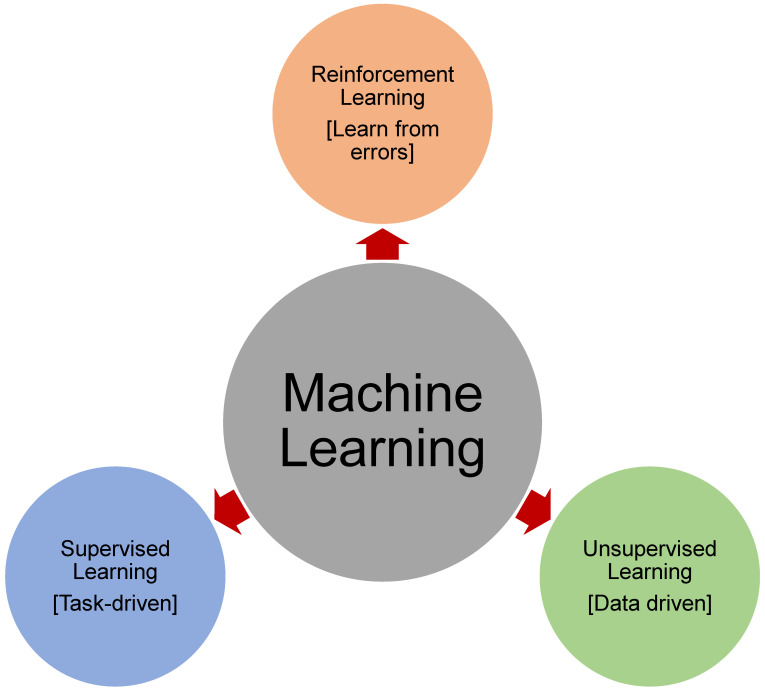
Categories of machine learning.

In general, ML is grouped into three classes (Figure 1): reinforcement learning, unsupervised learning, and supervised learning [57,58]. Supervised learning relates to regression and classification algorithms, which anticipate discrete or continuous results. In supervised approaches, the model is trained with known output data instances. The objective of unsupervised learning, on the other hand, is to detect relationships among datasets without specified descriptions, with the intention of grouping. Non-parametric models are sometimes known as unsupervised learning models [57]. Reinforcement learning, a less prevalent class of ML, is a form of trial-and-error learning that connects the space between unsupervised and supervised learning by identifying commonalities in the data and giving the right responses [59]. As a result of their adaptability and robust performance, ML approaches have attracted considerable interest in various civil engineering applications. They have been utilized mostly for the objectives of optimization and prediction [60,61]. In structural optimization, which tries to minimize the cost of a structure while providing a required performance, ML techniques are often used. ML approaches may be used to optimize the size, topology, and geometry of structural elements, so that the structure fulfils minimal design criteria [62]. In contrast, predictive models are designed to learn patterns from a given data sample and simplify them in order to make exact estimates. ML approaches have been used for a variety of issues in civil engineering, including structural health monitoring, geotechnics, fracture mechanics, etc. [63,64,65,66,67,68,69,70]. Estimations of various characteristics of conventional and advanced concretes, such as durability, thermal characteristics, and mechanical characteristics, have been extensively covered in previous studies [71,72,73,74].

### 1.4. Motivation and Significance of the the Study

As research on the use of ML to forecast concrete properties increases in response to advancing computational technologies, scientists are confronted with information restrictions that may stifle innovative research and academic collaboration. Consequently, it is crucial to develop and implement a system that helps academics to acquire essential knowledge from the most highly credible sources possible. Using a software program, a scientometric technique may help overcome this deficiency. In this study, we aim to perform a scientometric study of bibliographic records published on ML for concrete between 2001 and May of 2022. A scientometric assessment can achieve a quantifiable assessment of enormous amounts of bibliographic data by utilizing an appropriate software application. Conventional review studies lack the capacity to link disparate portions of the literature accurately and comprehensively. Scientific visualization, co-citations, and co-occurrence are among the highly complicated aspects of contemporary research [75,76,77,78]. The scientometric analysis revealed the sources with the most publications, keyword co-occurrence, the authors with the most papers and citations, the top articles in terms of citations, and the regions actively involved in research regarding ML applications for concrete. The Scopus search engine was used to obtain citation, bibliographic, abstract, keyword, funding, and other information from 1367 pertinent papers, which were then analyzed using the VOSviewer application. As a result of the graphical and statistical representation of researchers and countries, this study will assist scholars in developing collaborative endeavors and exchanging innovative concepts and techniques.

## 2. Review Strategy

**Figure 2:**
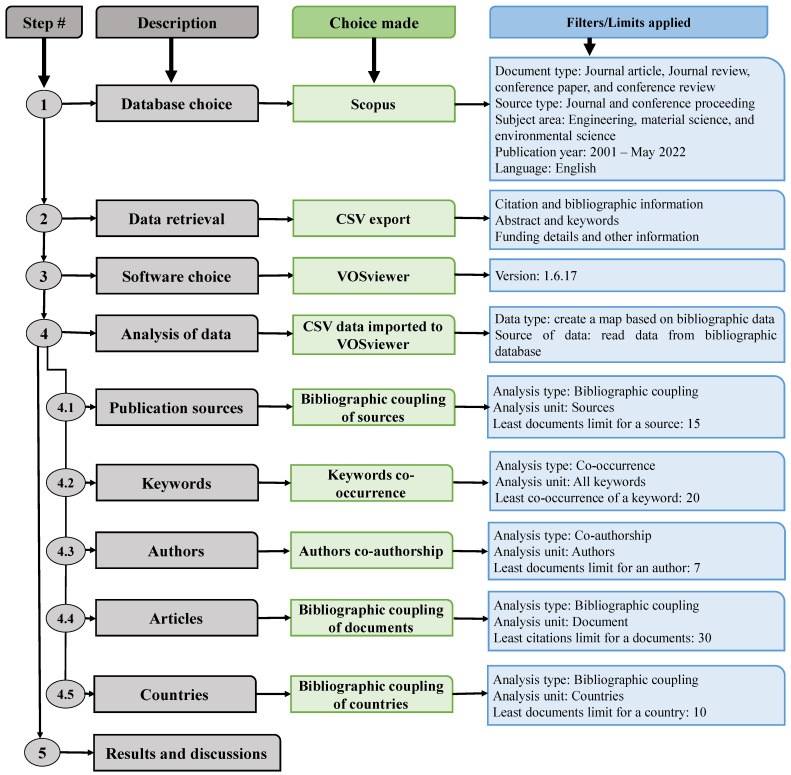
Flowchart of the study’s strategy, indicating various choices selected and limits applied during each step.

In this study, we conducted a scientometric analysis of bibliographic data [79,80,81] in order to quantify the numerous characteristics of the literature. Scientometric studies utilize scientific mapping, a technique established by academics for bibliometric data analysis [82,83]. Numerous articles have been published on the subject under study; thus, it was essential to utilize a credible search engine. Web of Science and Scopus are two extremely precise databases that are ideally fit for this purpose [84,85]. Scopus, which is highly recommended by academics [86,87], was used to collect bibliographic information for this study on ML for concrete. A May 2022 Scopus search for “machine learning for concrete” yielded 2468 results. Numerous filter settings were utilized to eliminate unnecessary papers. Figure 2 depicts a complete flowchart of the data retrieval, the analysis, and the numerous limits/filters applied during the analysis. The reason for selecting these filters at the data searching stage was to retrieve the relevant data within the domain of this study and maintain limits so that the data could be analyzed appropriately. For example, the subject areas of engineering and material and environmental science were selected, and the required language was English. In addition, the reason for choosing limits at different analysis steps such as publication sources, keywords, authors, etc., was to obtain results leading to better mapping and comparison. For example, the lowest document limit for a source was kept at 15. If the limit is set at lower values (e.g., 5), it results in more sources, and the map produced is hard to read. Additionally, other studies have been published using the same method [88,89,90,91]. Following the application of these filters to the Scopus database, 1367 records remained. The Scopus records were stored in a comma-separated values (CSV) format for further assessment using the relevant software. VOSviewer (version 1.6.17) was utilized to construct the scientific visualization and quantitative evaluation of the obtained material. VOSviewer is a freely accessible, open-source mapping tool that is generally employed in distinct study areas and well recommended by academics [92,93,94]. Consequently, the current study’s objectives were met by using VOSviewer. The resulting CSV file was loaded into VOSviewer, and further evaluation was conducted while maintaining data consistency and reliability. During the scientometric analysis, the publishing outlets, the most frequently occurring keywords, the researchers with the highest number of published articles and citations, the documents that received the most citations, and the state’s involvement were all evaluated. The multiple features, together with their interrelationships and co-occurrence are illustrated via maps, and the quantitative data are presented in tables.

## 3. Results Analysis

### 3.1. Subject Areas and Annual Articles Published

**Figure 3:**
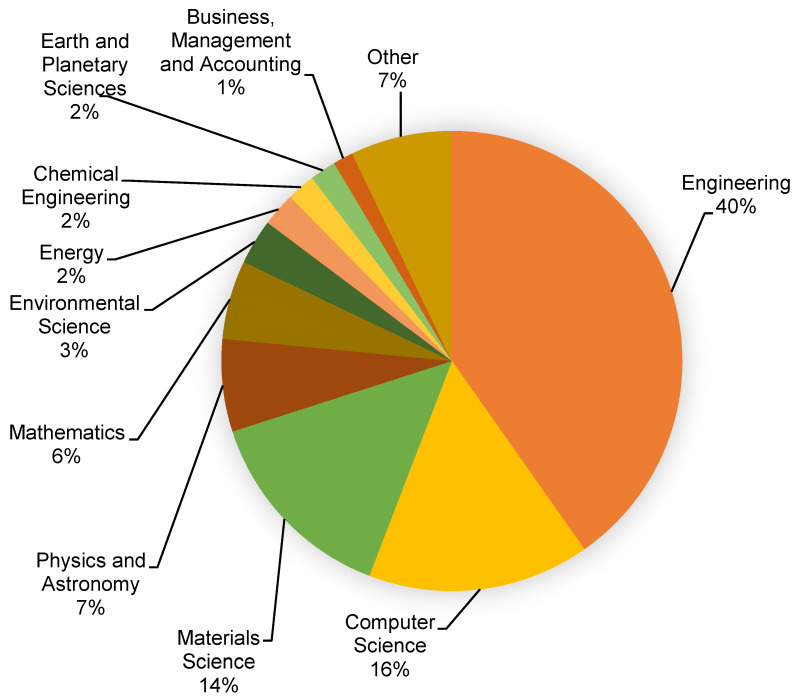
Relevant subject areas of documents.

**Figure 4:**
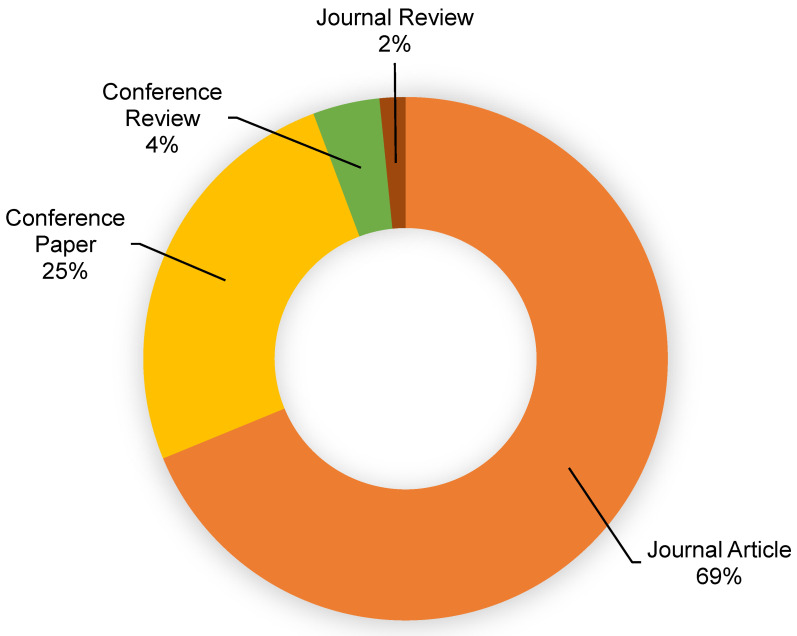
Article types published.

**Figure 5:**
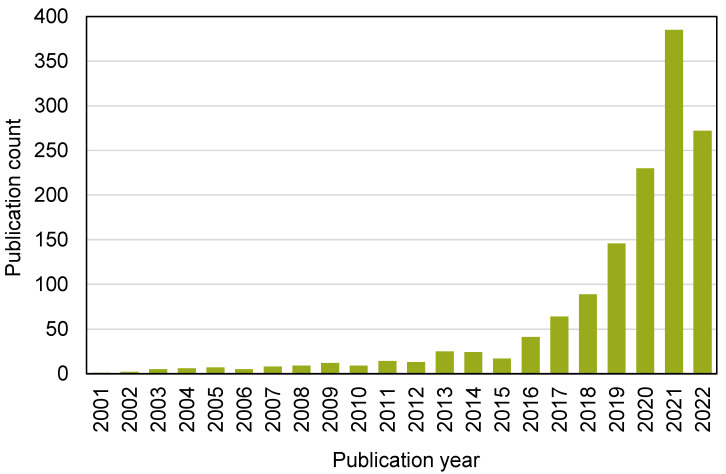
Annual publication trend for articles from 2001 to 2022 (May).

This assessment was performed using the Scopus analyzer to identify the most pertinent study fields. As seen in Figure 3, engineering, computer science, and materials science were found to be the top three document-generating disciplines, with about 40%, 16%, and 14% of documents, contributing a total of 70% of documents. In addition, the Scopus database was analyzed for the types of publications containing the sought phrase (Figure 4). Based on this assessment, journal papers, conference articles, conference reviews, and journal reviews comprised around 69%, 25%, 4%, and 2% of all materials, respectively. Figure 5 depicts the annual development of articles published in the various study fields from 2001 to May 2022. The year limit on the subject research field was set to start from 2001. Up to 2015, there was modest growth in the number of publications in the field of ML for concrete research, with an average of around 10 papers each year. Subsequently, there was a significant increase in the number of articles, with an average of around 159 articles each year between 2016 and 2021 and 385 articles in 2021. The number of publications is increasing each year, and in the current year, the number of publications in the research area is 272 so far (May 2022). It is fascinating to see that researchers are focusing their attention on the use of contemporary tools such as ML for estimating the properties of construction materials. This will provide the building sector with more rapid and cost-efficient methods by reducing the need for experimental procedures.

### 3.2. Publication Sources

**Figure 6:**
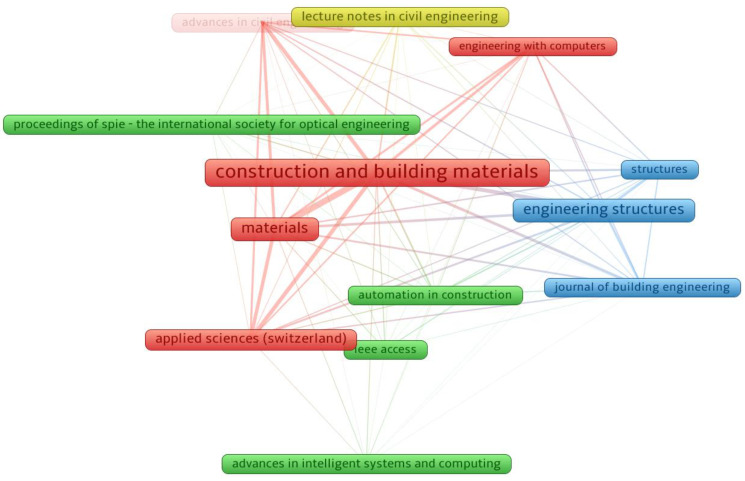
Systematic map of journals that published a minimum of 10 documents.

An evaluation of publication outlets (journals) was performed on the data using VOSviewer. A minimum of 15 papers per source was stipulated, and 13 of the 541 publication sources satisfied this requirement. Table 1 displays the publishing outlets that released at least 10 publications on ML for concrete up to May 2022, along with the number of citations received within that time frame. “*Construction and Building Materials (CONBUILDMAT)*”, “*Materials*”, and “*Engineering Structures*” were found to be the top publication journals with 94, 52, and 48 papers, respectively. Furthermore, the same three sources obtained the greatest number of citations between 2001 and May 2022, with “*CONBUILDMAT*” obtaining 1677, “*Engineering Structures*” receiving 602, and “*Materials*” receiving 441 citations. This examination could provide the groundwork for forthcoming scientometric evaluations in ML research for concrete. Additionally, previous conventional review studies were unable to produce systematic graphs. Figure 6 shows a visualization of the sources publishing at least 15 articles. The frame dimension is related to the outlet’s influence on the present study field, based on document count; a bigger frame size indicates a greater impact. As an illustration, “*CONBUILDMAT*” has a larger frame than the others, indicating that it is a journal of great significance in the present research field. Four clusters were formed, characterized by a distinct color on the map (blue, red, yellow, and green). Clusters were developed based on the extent of the research outlet or the frequency with which they were co-cited in comparable articles [95]. The VOSviewer grouped journals according to their co-citation tendencies with regard to published articles. For example, the red cluster comprises five journals that were co-cited many times in the same work. In addition, the links between closely located frames (sources) in a group/cluster are greater than those between widely spread frames. For example, “*CONBUIDMAT*” correlates more strongly with “*Materials*” than with “*Computer-Aided Engineering*”.

### 3.3. Keywords

**Figure 7:**
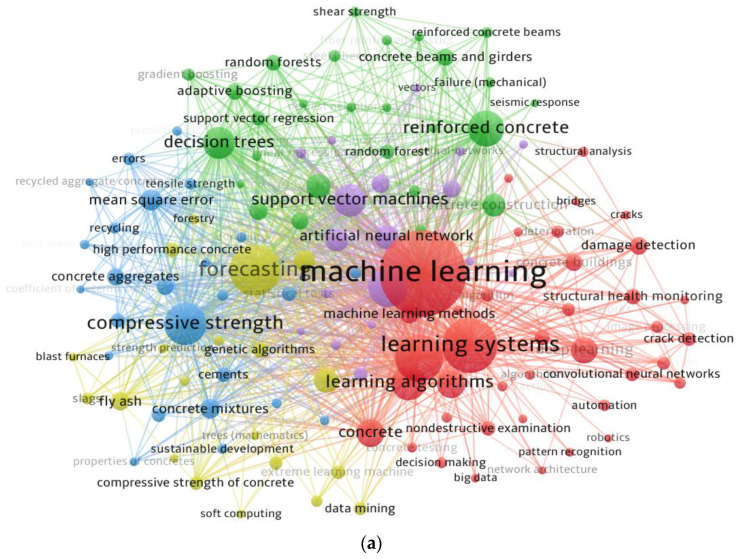

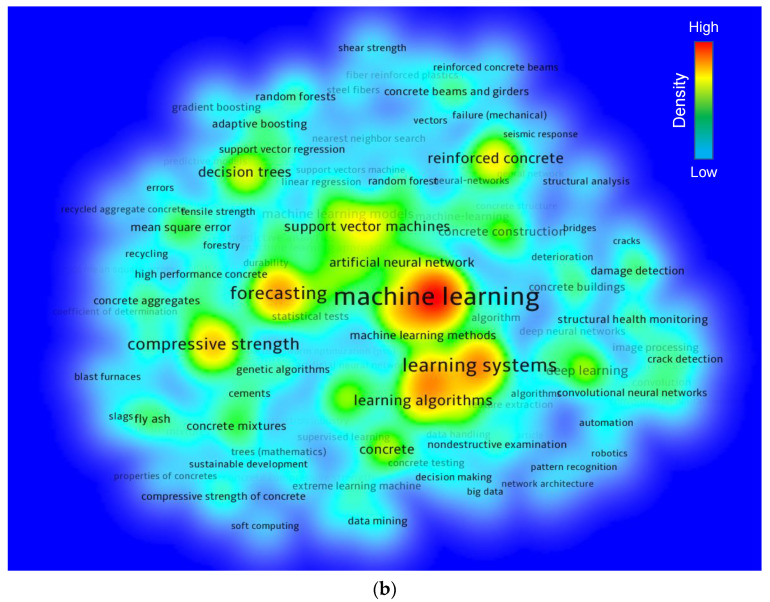
Systematic map of keywords: (**a**) scientific mapping; (**b**) density.

Keywords are significant in research since they distinguish and emphasize the basic subjects of the study domain [96]. The minimum repetition requirement for a keyword was set at 20, and 129 of the 9872 keywords were preserved. Table 2 records the leading 30 keywords most frequently used in published studies on the subject. The 5 most often occurring terms in the topic study field were machine learning, learning systems, forecasting, concretes, and compressive strength. According to the keyword analysis, ML has mostly been used to predict concrete properties, particularly compressive strength. Figure 7 shows a systematic graph of keywords based on co-occurrences and connections, with densities proportional to their occurrence frequency. In Figure 7a, a keyword’s node size signifies its frequency, while its position suggests its co-occurrence in articles. In addition, the graph shows that the top keywords have wider nodes than the rest, signifying that these are essential ML keywords in a real investigation. The graph highlights clusters in a manner that shows their co-occurrence in a variety of published documents. The color-encoded grouping is determined by the co-occurrence of several keywords in publications. Five clusters are represented by different colors (green, red, blue, purple, and yellow) in Figure 7a. As observed in Figure 7b, distinct colors represent differing keyword density concentrations. The colors red, yellow, green, and blue are arranged according to their density strengths, with red representing the highest density concentration and blue representing the lowest. Machine learning, learning systems, forecasting, and other prominent keywords are shown in the red group, indicating a greater density of occurrences. This finding will help ambitious researchers select keywords that ease the discovery of published papers on a specific topic.

### 3.4. Authors

**Figure 8:**
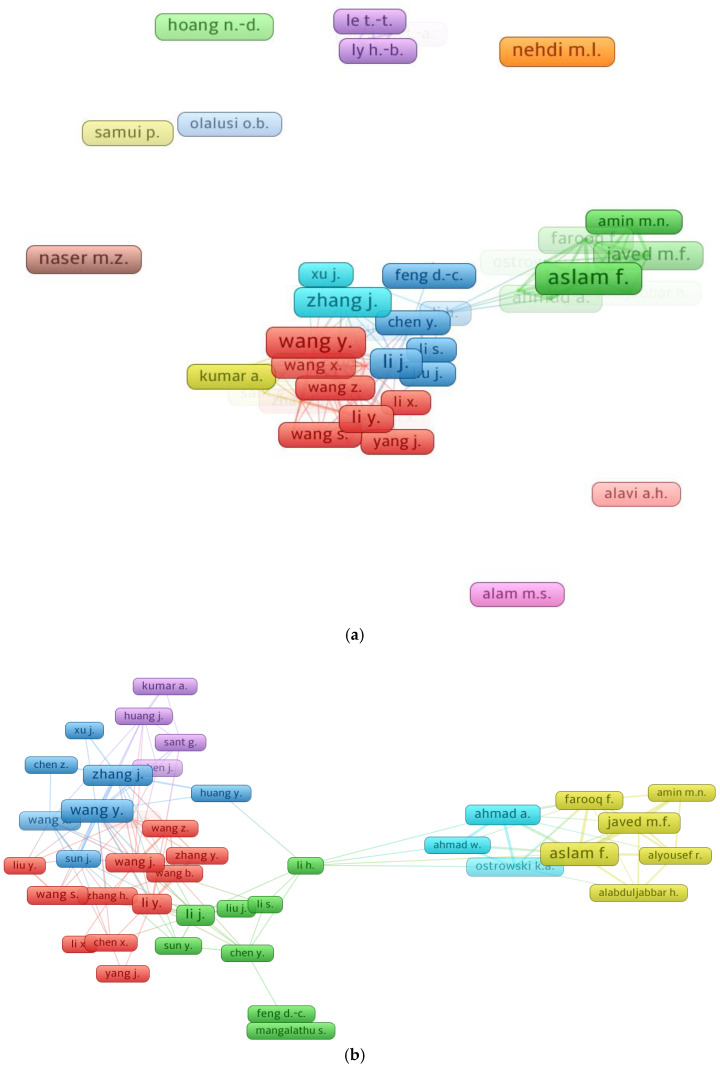
Systematic map of researchers: (**a**) authors with a least 7 articles; (**b**) connected authors.

Citations indicate a scientist’s impact in a particular field of research [97]. The threshold for the least number of papers for a researcher was set at 7, and 53 out of 3536 researchers satisfied this requirement. The authors with the most articles and citations in the field of ML for concrete, as assessed from the bibliographic data using VOSviewer, are included in Table 3. Each author’s average number of citations was determined by dividing the total citations by the total number of articles. It is complicated to assess the effectiveness of a scientist when all parameters (such as the quantity of documents, overall citations, and average citations) are taken into account. Alternatively, the researcher’s ranking can be evaluated separately for each component, i.e., the number of documents, the number of overall citations, and the average number of citations. The analysis revealed that Aslam F. was the most prolific researcher, with 25 publications, followed by Wang Y. with 22 and Nehdi M.L. with 19 publications. In terms of total citations, Mangalathu S. led the field with 385, Wang Y. was second with 351, and Nehdi M.L. was third with 327 overall citations in the present research domain. In addition, when the average numbers of citations were compared, the authors were ranked with Mangalathu S. at the top with nearly 48 citations, Alyousef R. in second place with about 24, and Liu J., Li S., and Feng D.-C. in third place, each having approximately 48 average citations. Figure 8 depicts the association between writers with at least 7 publications and the most notable authors. Figure 8a depicts the scientific mapping of scholars who have contributed at least 7 papers to the current field of study. Figure 8b depicts the largest group of related writers based on citations, which consists of 40 of the 53 authors. This investigation indicated that the majority of researchers working on ML applications for concrete are linked via citations.

### 3.5. Documents

**Figure 9:**
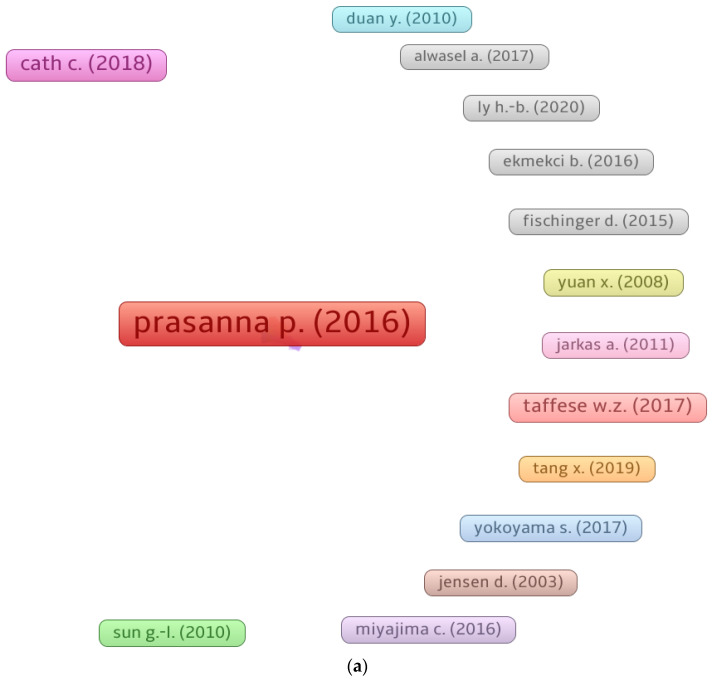

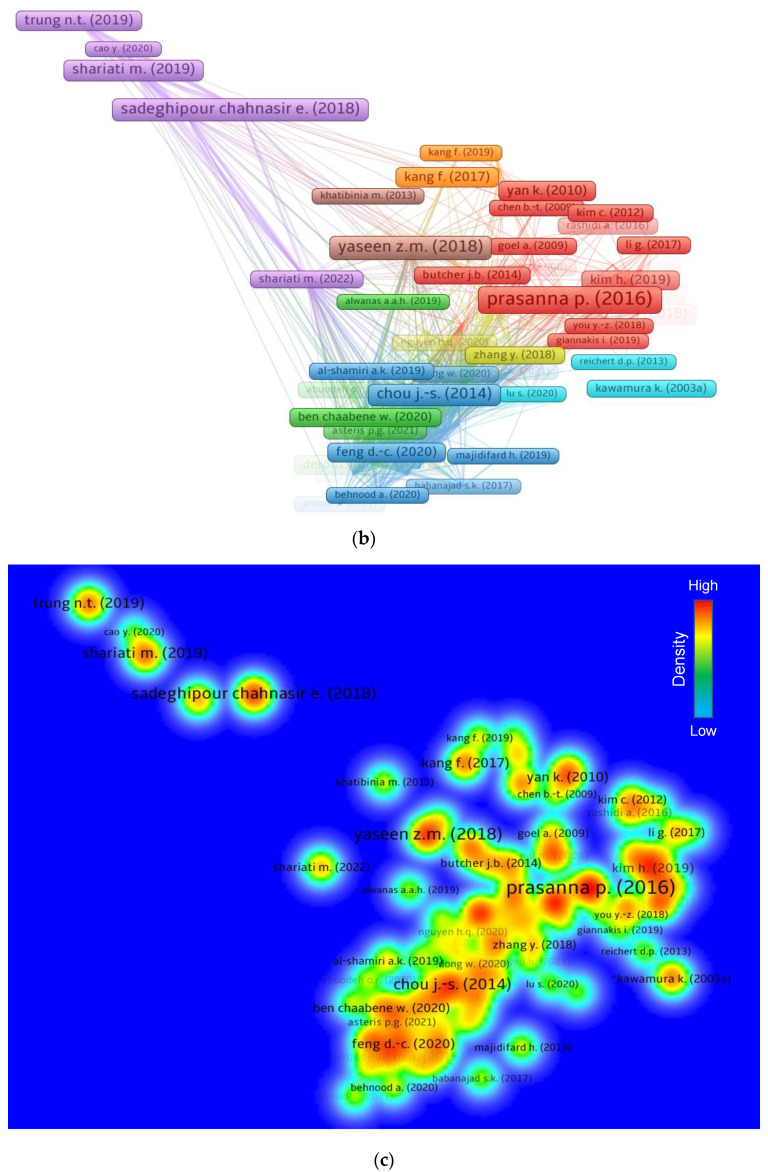
Systematic map of published documents up to May 2022: (**a**) documents with at least 30 citations; (**b**) linked documents based on citations; (**c**) density of connected documents.

The number of citations a document obtains signifies its influence in a certain research domain. In their respective study domains, the papers with the most citations are regarded as pioneering. The least number of citations for a document was set at 30, and 125 out of 1367 papers met this threshold. In Table 4, the top 5 articles in the field of ML for concrete based on citations are included, along with their authors and citation counts. The study “Automated Crack Detection on Concrete Bridges” by Prasanna P. [98] obtained 224 citations. Rafiei M.H. [99] and Chou J.-S. [100] received 184 and 174 citations, respectively, for their articles and were positioned in the leading three. However, up to May 2022, only 14 papers had acquired more than 100 citations. Figure 9 shows the scientific visualization of articles on the basis of citations and the density concentration of these articles in the domain of the present study. The map of papers with at least 30 citations is shown in Figure 9a. Figure 9b shows that 111 of 125 publications were related by citations, as determined by the VOSviewer analysis. In addition, the density mapping (Figure 9c) demonstrates the increased density concentration of the top articles, based on citations.

### 3.6. Countries

**Figure 10:**
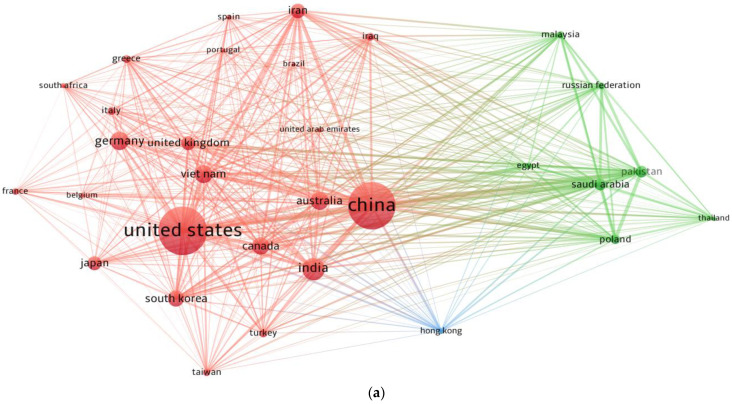

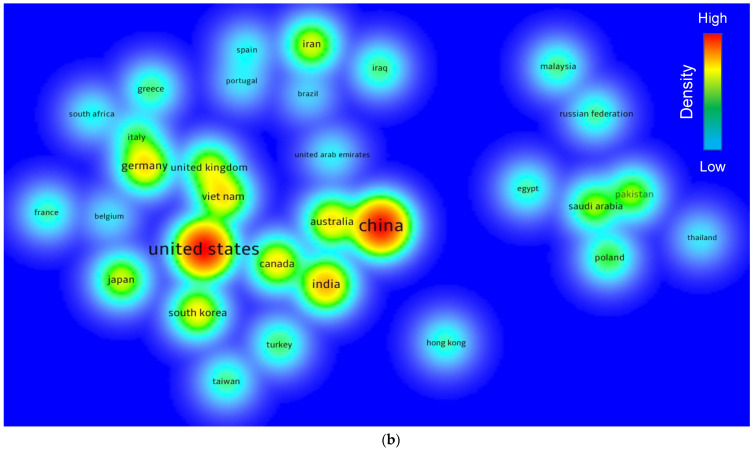
Systematic map of countries that presented a minimum of 10 articles from 2001 to May 2022: (**a**) network map; (**b**) density map.

Numerous countries have presented more documents in the present research area than others, and they plan to continue their contributions. The systematic map was constructed so that readers may examine the regions performing ML applications research for predicting concrete properties. The minimum number of documents a country could possess in order to be included was set at 10, and 31 countries satisfied this threshold. The countries included in Table 5 produced a minimum of 10 documents on the current topic of research. The United States, China, and India had the greatest number of papers, with 298, 289, and 110 documents, respectively. In addition, papers from the United States received 4260 citations, followed by papers from China with 2732 citations. Papers from Vietnam received 1633 citations. Figure 10 shows the systematic map and the density strength of countries linked by citations. In Figure 10a, the size of a node is proportional to a country’s impact on the topic studied, based on the number of articles. As seen in Figure 10b, the countries with the greatest levels of participation had a greater density. The graphical depiction and quantitative record of the participating countries will assist young scientists in creating scientific partnerships, launching collaborative ventures, and exchanging creative approaches and concepts. Scholars from countries concerned with advancing research on ML applications for concrete can collaborate with other professionals in the field and benefit from their knowledge.

## 4. Discussions and Recommendations for Future Work

This systematic review performed statistical analysis and mapping of the bibliographic data available on the applications of ML for predicting concrete properties. Previous manual review studies lacked the capacity to link disparate areas of the literature completely and precisely. This analysis identified the sources of publications (journals) that published the most documents, the keywords most often used in publications, the documents and researchers with the highest numbers of citations, and the countries that are vigorously engaged in ML applications for concrete research. According to the keyword analysis, ML has been utilized mostly to forecast concrete properties, particularly compressive strength. In addition, the literature and the linkages based on citations were used to identify the highly committed and participating countries, based on publication count. The graphical representation and quantitative analysis of the participating countries and researchers will help young scientists form scientific partnerships, establish joint ventures, and share advanced methods and concepts. Scholars from countries concerned with expanding the research on the applications of ML for concrete can collaborate with other professionals in the discipline and benefit from their expertise.

We are on the verge of a fourth industrial revolution, in which data-driven smart approaches, robotics, additive manufacturing, cloud computing, the Internet of Things, and other developing tools will merge the physical, biological, and digital realms. The building industry is lagging behind in seizing the openings presented by the world’s fast transformation. The engineering properties of building materials and structures predicted by ML have applications in propagative smart design. Several knowledge gaps exist that must be filled before structural engineers can imitate procedures employed in mechatronics, robotics, and other sophisticated domains. Table 6 lists the various types of ML techniques employed to estimate the properties of materials, the numbers of inputs and data samples used to run the models, and the best ML techniques as recommended by the literature. Most of the previous studies suggested increasing the number of inputs to include the chemical composition of raw ingredients and the environmental conditions (humidity and temperature). In addition, increasing the number of data samples via further experimental tests might enhance the performance of ML models in terms of real and accurate prediction [52,103,104,105]. Hence, practical applications of ML in the building sector require further in-depth investigations, in order to propose guidelines for ML’s applicability.

## 5. Conclusions

The purpose of this study was to undertake a scientometric assessment of the available literature on machine learning (ML) applications for concrete, in order to evaluate various metrics. The database Scopus was searched, 1367 related articles were found, and the data were evaluated using the VOSviewer application. The following conclusions were obtained from this investigation:

- An assessment of publication journals including articles on ML for concrete research revealed that “*CONBUILDMAT*”, “*Materials*”, and “*Engineering Structures*” were the top three sources, with 94, 52, and 48 publications, respectively. In terms of total citations, the top three publishing sources were “*CONBULDMAT*” with 1677, “*Engineering Structures*” with 602, and “*Materials*” with 441.
- A keyword analysis of the topic research field revealed that machine learning, learning systems, forecasting, concretes, and compressive strength were the five terms occurring most often. The keyword analysis found that machine learning had mostly been used to forecast concrete properties, particularly compressive strength.
- The author analysis found that just 53 authors had published at least 7 articles on ML for concrete research. The leading authors were categorized according to their document count, overall citations, and average citations. With 25 publications, Aslam F. was the most prolific author, followed by Wang Y. with 22 and Nehdi M.L. with 19 papers. In terms of total citations, Mangalathu S. had the highest number, with 385, followed by Wang Y. with 351 and Nehdi M.L. with 327 total citations. Moreover, when comparing the average number of citations, the following writers stood out: Mangalathu S. with roughly 48 citations, Alyousef R. with nearly 24, and Liu J., Li S., and Feng D.-C., each with an average number of citations of about 48.
- An evaluation of articles offering data on ML applications for concrete revealed that the study by Prasanna P. [98] on “Automated Crack Detection on Concrete Bridges” received 224 citations. Rafiei M.H. [99] and Chou J.-S. [100] obtained 184 and 174 citations, respectively, for their studies and were among the top three. In addition, as of May 2022, only 14 papers had received more than 100 citations in the topic field.
- Based on their engagement in ML concrete research, the main countries were identified, and it was found that only 31 countries had produced at least 10 publications. The United States, China, and India produced 298, 289, and 110 papers, respectively. In addition, the papers from the United States received 4260 citations, those from China received 2732 citations, and those from Vietnam received 1633 citations.
- These revolutionary techniques will aid the building sector by enabling the creation of efficient and economical methods for evaluating the properties of materials. In addition, the adoption and application of a material in the building sector will be expedited by encouraging computational methods.
- The prevalence of ML applications is forecast to increase as the Internet of Things, big data, and automated systems continue to dominate the industrial sector in the next decades.
- To improve the performance of ML models, it is recommended that a greater number of input factors should be employed, such as the chemical composition of raw components and the environmental conditions (humidity and temperature). In addition, increasing the number of data samples through additional experimental testing may improve the performance of ML models in terms of real and precise predictions.

## Figures and Tables

**Table 1:** List of journals publishing a minimum of 15 documents in the subject domain from 2001 to May 2022.

S/N	Source Name	Total Publications	Total Citations
1	*Construction and Building Materials*	94	1677
2	*Materials*	52	441
3	*Engineering Structures*	48	602
4	*Applied Sciences (Switzerland)*	35	258
5	*Lecture Notes in Civil Engineering*	30	4
6	*Proceedings of SPIE—the International Society for Optical Engineering*	23	32
7	*Advances in Intelligent Systems and Computing*	22	13
8	*Structures*	21	95
9	*Advances in Civil Engineering*	20	220
10	*Journal of Building Engineering*	18	185
11	*IEEE Access*	18	108
12	*Automation in Construction*	17	422
13	*Engineering with Computers*	15	344

**Table 2:** List of the 30 most commonly used keywords in the studies of ML applications for concrete.

S/N	Keyword	Occurrences
1	Machine learning	761
2	Learning systems	378
3	Forecasting	334
4	Concretes	301
5	Compressive strength	252
6	Neural networks	225
7	Reinforced concrete	196
8	Learning algorithms	185
9	Support vector machines	169
10	Decision trees	162
11	Artificial intelligence	137
12	Concrete	134
13	Deep learning	120
14	Machine learning techniques	109
15	Artificial neural network	108
16	Machine learning models	107
17	Concrete construction	100
18	Regression analysis	97
19	Prediction	88
20	Mean square error	85
21	Concrete mixtures	79
22	Machine learning methods	72
23	Support vector machine	71
24	Predictive analytics	69
25	Fly ash	68
26	Damage detection	67
27	Machine-learning	66
28	Concrete buildings	61
29	Machine learning approaches	61
30	Concrete aggregates	60

**Table 3:** List of researchers having at least 7 articles in the subject research domain from 2001 to May 2022.

S/N	Researcher Name	Total Publications	Overall Citations	Average Citations
1	Aslam F.	25	297	12
2	Wang Y.	22	351	16
3	Nehdi M.L.	19	327	17
4	Li J.	18	295	16
5	Zhang J.	18	282	16
6	Javed M.F.	17	147	9
7	Naser M.Z.	16	129	8
8	Ahmad A.	15	159	11
9	Li Y.	15	66	4
10	Farooq F.	13	258	20
11	Hoang N.-D.	13	249	19
12	Wang J.	13	90	7
13	Samui P.	12	182	15
14	Ostrowski K.A.	12	115	10
15	Wang X.	12	66	6
16	Wang S.	12	52	4
17	Le T.-T.	11	222	20
18	Ly H.-B.	11	151	14
19	Kumar A.	10	141	14
20	Alyousef R.	9	215	24
21	Feng D.-C.	9	195	22
22	Zhang Y.	9	187	21
23	Yang J.	9	40	4
24	Mangalathu S.	8	385	48
25	Chen Y.	8	136	17
26	Alavi A.H.	8	118	15
27	Tran V.Q.	8	106	13
28	Zhang Z.	8	64	8
29	Chen X.	8	51	6
30	Ahmad W.	8	46	6
31	Wang Z.	8	32	4
32	Liu J.	7	157	22
33	Li S.	7	152	22
34	Chen Z.	7	146	21
35	Huang J.	7	133	19
36	Sant G.	7	127	18
37	Amin M.N.	7	117	17
38	Huang Y.	7	108	15
39	Xu J.	7	103	15
40	Thai H.-T.	7	97	14
41	Alabduljabbar H.	7	86	12
42	Chen J.	7	85	12
43	Sun Y.	7	79	11
44	Nguyen T.-A.	7	71	10
45	Sun J.	7	61	9
46	Olalusi O.B.	7	43	6
47	Zhang H.	7	43	6
48	Marani A.	7	37	5
49	Li X.	7	34	5
50	Wang B.	7	29	4
51	Alam M.S.	7	14	2
52	Liu Y.	7	14	2
53	Li H.	7	10	1

**Table 4:** List of top 5 documents in terms of citations received up to May 2022.

S/N	Article	Title	Total Number of Citations Received
1	Prasanna P. [98]	Automated Crack Detection on Concrete Bridges	224
2	Rafiei M.H. [99]	A novel machine learning-based algorithm to detect damage in high-rise building structures	184
3	Chou J.-S. [100]	Optimizing the prediction accuracy of concrete compressive strength based on a comparison of data-mining techniques	174
4	Yaseen Z.M. [101]	Predicting compressive strength of lightweight foamed concrete using extreme learning machine model	170
5	Sadeghipour Chahnasir E. [102]	Application of support vector machine with firefly algorithm for investigation of the factors affecting the shear strength of angle shear connectors	165

**Table 5:** List of countries that presented at least 10 papers in the subject research domain from 2001 to May 2022.

S/N	Country	Documents Published	Overall Citations
1	United States	298	4260
2	China	289	2732
3	India	110	725
4	Germany	84	479
5	Vietnam	82	1633
6	Australia	81	1066
7	Canada	77	824
8	South Korea	72	1063
9	Iran	62	1188
10	United Kingdom	58	578
11	Japan	58	468
12	Saudi Arabia	48	477
13	Pakistan	47	404
14	Poland	35	373
15	Italy	29	236
16	Turkey	29	217
17	Iraq	27	610
18	Greece	27	170
19	Malaysia	26	658
20	Taiwan	25	638
21	Russian Federation	24	59
22	France	22	237
23	Egypt	22	148
24	Hong Kong	21	175
25	South Africa	16	68
26	Spain	15	280
27	United Arab Emirates	14	99
28	Thailand	13	68
29	Belgium	12	170
30	Portugal	12	117
31	Brazil	10	18

**Table 6:** Types of machine learning techniques used in previous studies.

Ref.	Material Type	Properties Predicted	ML Techniques Employed	No. of Input Parameters	Data Points	Best ML Technique Recommended
[49]	Recycled aggregate concrete	Compressive strength	Decision tree, gradient boosting, and bagging regressor	8	638	Bagging regressor
[106]	Concrete-filled steel tubes	Ultimate axial capacity	GEP	6	227	-
[107]	Geopolymer concrete	Compressive strength	Decision tree, GEP, bagging regressor, and random forest	9	371	Bagging regressor
[105]	High-performance concrete	Compressive strength	Decision tree, multilayer perceptron neural network, support vector machine, extreme gradient boosting, AdaBoost, bagging regressor, and random forest	8	1030	Random forest and decision tree with bagging
[53]	Recycled aggregate concrete	Splitting tensile strength	GEP, ANN, and bagging regressor	9	166	Bagging regressor
[103]	Rice husk ash concrete	Compressive strength	GEP and random forest	6	192	GEP
[52]	Recycled aggregate concrete	Compressive and flexural strength	Gradient boosting and random forest	12	638	Random forest
[108]	Geopolymer concrete	Compressive strength	Decision tree, random forest, and AdaBoost	9	363	AdaBoost and random forest
[109]	Recycled aggregate concrete	Compressive and splitting tensile strength	AdaBoost and decision tree	9	344	AdaBoost
[110]	Geopolymer concrete	Compressive strength	Decision tree, bagging regressor, and AdaBoost	9	154	Bagging regressor
[51]	High-performance concrete	Compressive strength	Support vector machine, AdaBoost, and random forest	7	1030	Random forest
[35]	High-performance concrete	Compressive strength	Decision tree, GEP, AdaBoost, and bagging regressor	8	1030	Bagging regressor
[8]	Recycled aggregate concrete	Compressive strength	GEP and ANN	9	344	GEP
[111]	Fly-ash-based concrete	Compressive strength	GEP, ANN, decision tree, and bagging regressor	7	98	Bagging regressor
[104]	Fly-ash-based concrete	Compressive strength	GEP, decision tree, and bagging regressor	8	270	Bagging regressor
[112]	Waste-material-based concrete	Surface chloride concentration	GEP, decision tree, and ANN	12	642	GEP
[113]	High-strength concrete	Compressive strength	GEP and random forest	5	357	Random forest

ANN: artificial neural network; GEP: gene expression programming.

## Data Availability

The data used in this research have been properly cited and reported in the main text.
